# A guide to performing a peer review of randomised controlled trials

**DOI:** 10.1186/s12916-015-0471-8

**Published:** 2015-11-02

**Authors:** Chris Del Mar, Tammy C. Hoffmann

**Affiliations:** Centre for Research in Evidence-Based Practice, Faculty of Health Sciences and Medicine, Bond University, Gold Coast, Queensland, 4229 Australia

**Keywords:** CONSORT statement, Editorial responsibilities, Medical journal publishing, Peer review, Randomised controlled trial

## Abstract

Peer review of journal articles is an important step in the research process. Editors rely on the expertise of peer reviewers to properly assess submissions. Yet, peer review quality varies widely and few receive training or guidance in how to approach the task.

This paper describes some of the main steps that peer reviewers in general and, in particular, those performing reviewes of randomised controlled trials (RCT), can use when carrying out a review. It can be helpful to begin with a brief read to acquaint yourself with the study, followed by a detailed read and a careful check for flaws. These can be divided into ‘major’ (problems that must be resolved before publication can be considered) and ‘minor’ (suggested improvements that are discretionary) flaws. Being aware of the appropriate reporting checklist for the study being reviewed (such as CONSORT and its extensions for RCTs) can also be valuable.

Competing interests or prejudices might corrode the review, so ensuring transparency about them is important. Finally, ensuring that the paper’s strengths are acknowledged along with a dissection of the weaknesses provides balance and perspective to both authors and editors. Helpful reviews are constructive and improve the quality of the paper. The proper conduct of a peer review is the responsibility of all who accept the role.

## Background

Peer review of journal articles is an important process in research. It is part of the underlying engine that aims to sort out which papers will be published and what modifications are needed before this occurs.

It is easy to assume that reviewers’ duties and editors’ objectives are identical: we all want to see that good papers are accepted and flawed ones rejected, and that all are papers improved by the review process. In a study of over 200 reviewers of randomised controlled trials (RCTs) in high impact medical journals, reviewers ranked activities such as ‘evaluating the risk of bias’, and ‘checking that the conclusions were consistent with the results’, top [1]. However, the 171 participating editors ranked these much lower, instead wanting to simply know whether the reviewers ‘did or did not recommend publication’ and ‘whether this was an important topic’ [1].

Editors rely on the expertise of their peer reviewers to provide the necessary background (typically content and/or methodological) to properly assess submissions. While there are imperfections in the peer review system and some doubts about the impact of peer review [2, 3], it is a system that is used universally. However, the quality of peer review varies enormously and few reviewers receive training in how to do it and may not be aware of some of the elements to consider or when new to reviewing, how to approach the task. In this paper, we describe some of the main steps that peer reviewers of research papers in general, and RCTs in particular, can use to guide their review.

## General steps – for all research articles

### Acquaint yourself with the paper

It is a good idea to read the paper soon after your commission to referee it. Leaving it to the last minute risks introducing a delay if you discover a problem which means that you cannot complete it (for example, a conflict of interest that only emerges after a detailed read or addressing an area in which you have no expertise). In any case, a rapid read allows you to get a fast grasp of the content to prepare you for the full task. Breaking the research study into its component parts can help you to understand the main elements of the study. For those familiar with doing this step in critical appraisal, the example component parts for most RCTs would be: Participants/Patient, Intervention, Comparator, and Outcomes (or PICO). For studies that are not RCTs, many of these components are still relevant – for example, Participants/Problem/Population, Issue/Index (if an observational study), and Outcomes. Identifying what the study’s question is can help to initially orient you to the paper and consider whether the most appropriate study design was used to answer the question posed by the researchers.

Many journals ask their reviewers to classify remaining flaws into ‘major’ (defined as requiring satisfactory resolution before publication can be considered) and ‘minor’ (discretionary, suggestions that might improve the paper, but could be ignored). This is a useful classification system to follow anyway, even if not a requirement of the journal.

### Is the quality good enough? Check for major flaws

Among the things that editors have to decide, one of the most essential is whether a paper is fatally flawed. This means that, whatever else – even if a fascinating topic, and beautifully crafted in breathless prose – a critical problem may mean it cannot be published, even with complete overhaul. The word ‘fatal’ implies no hope of resuscitation. Sometimes what appears to be a fatal flaw may actually be inadequate reporting (for example, not reporting ethical approval or trial registration), in which case, stop the review until this is addressed with the authors of the paper (through the editorial office of course – never directly with the authors). The Editor/editorial office may ask the authors to provide more information to resolve the impasse. Some journals screen for certain types of fatal flaws before sending papers for review; others do not. If the issue is not inadequate reporting and there is truly a fatal flaw, stop. There is nothing else necessary to comment on. Focus your report on that fatal flaw; however, do mention that you did not review the remainder of the paper. Your responsibility as a peer reviewer ends when you describe how it is not possible to repair this paper. Bear in mind that what is a ‘fatal’ flaw can be quite subjective and will vary between journals, editors, and reviewers. What some believe is fatal, others may see as a major flaw that is reparable in some way.

Major flaws are serious flaws that must be addressed before publication can be considered appropriate. Some examples of major flaws, that are applicable to many study designs, are provided in Box 1.

### Major flaws in RCTs

There are special requirements for the conduct and reporting of RCTs, and peer reviewers should carefully check that these requirements have been met. Unfortunately, peer reviewers are not usually good at detecting these [4]. In one study, a journal asked 607 peer reviewers to participate and gave them three (previously published) RCTs which were altered so that, after removing the original authors’ names and any other identifying features, nine major errors were deliberately added [5]; reviewers were able to identify a mean of only 2.6 errors. There have been calls for innovations in providing training to would-be reviewers of RCTs before they can enter a register of ‘RCT reviewers’ that journals could draw upon [6]. Interventions to improve the quality of peer review have had mixed results when evaluated in RCTs [5, 7, 8]. The most recent trial suggested, although not conclusively, that conducting an additional review based on reporting guidelines might result in small improvements in paper quality [8].

Critically appraising the RCT to determine its risk of bias, and where necessary, whether this has been appropriately acknowledged, is important. Reviewers who are less experienced with critical appraisal might find using the mnemonic RAMbo useful to assess key types of potential bias: **R**andomised (was there random allocation, concealed allocation, and baseline similarity of groups?); **A**ttrition (was there adequate follow-up, intention-to-treat analysis, and, aside from the experimental intervention, were the groups treated equally?); and **M**easurement (was it done by **b**linded assessors, or were **o**bjective measures used?).

It is good practice to undertake an extra step in reviewing RCTs, to check either the published protocol, or its registry entry, to establish whether all measured outcomes were reported. The statistical analyses of RCTs sometimes require specialized expertise. If you are unsure whether the analyses have been performed or interpreted correctly, do not be afraid to alert the editor to this and report that a statistical review is also required. Journals often have statistical reviewers they can call on, and alerting the Editors to a specific need may be very helpful.

### What else could be improved? Check for minor flaws

Examples of minor flaws include writing that is clumsy; lack of clarity in some of the argument (usually in the Background and/or Discussion); minor details missing or inadequately reported; tables or figures that duplicate the text or, conversely, are not appropriately referred to; unclear tables or figures; or referencing errors. Tables and figures should typically be self-contained. This means that it should be possible to understand them without reference to the text.

**Fig. 1:**
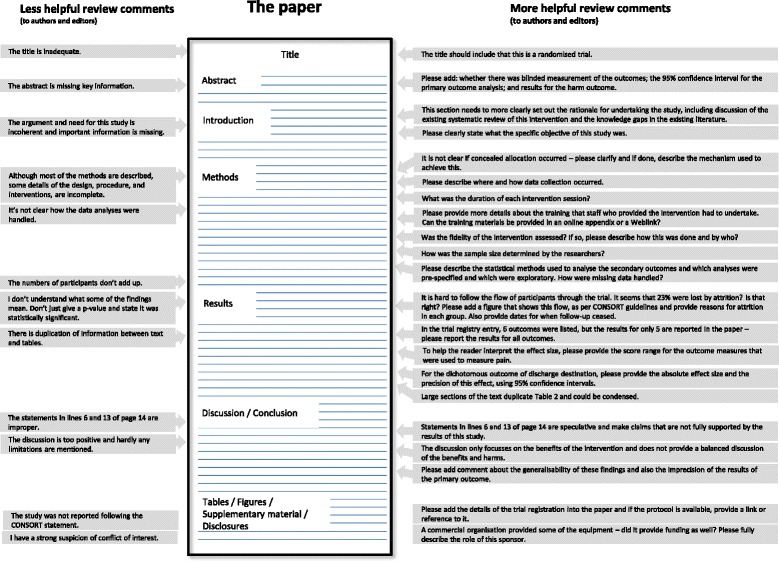
Examples of ‘less helpful’ and ‘more helpful’ peer review comments on a hypothetical RCT paper

Sometimes you might want to debate a statement that you disagree with. Here, we enter a more subjective territory. Some of your comments may be very helpful – and authors may well thank the reviewer for the suggestion (this is common), or refute it with some well-constructed arguments (just as common!). Nevertheless, it is worth labelling your concerns as those suggested improvements which you think the authors need to take up before publication, and those which are best left to the discretion of the authors and editors. Figure 1 provides examples of ‘less helpful’ and ‘more helpful’ reviewer’s comments, on a number of major and minor flaws, for a hypothetical RCT.

### Using reporting guidelines

Where better to find guidelines about which items are essential for each study design than those intended for use by authors? Reporting guidelines can be used to help inform peer review [9]. There are reporting guidelines for many of the major study designs. For example, there are reporting statements for RCTs, systematic reviews, observational studies, diagnostic studies, case reports, economic evaluations, and even for protocols of studies. A complete list of reporting guidelines is maintained by the EQUATOR Network [10].

**Fig. 2:**
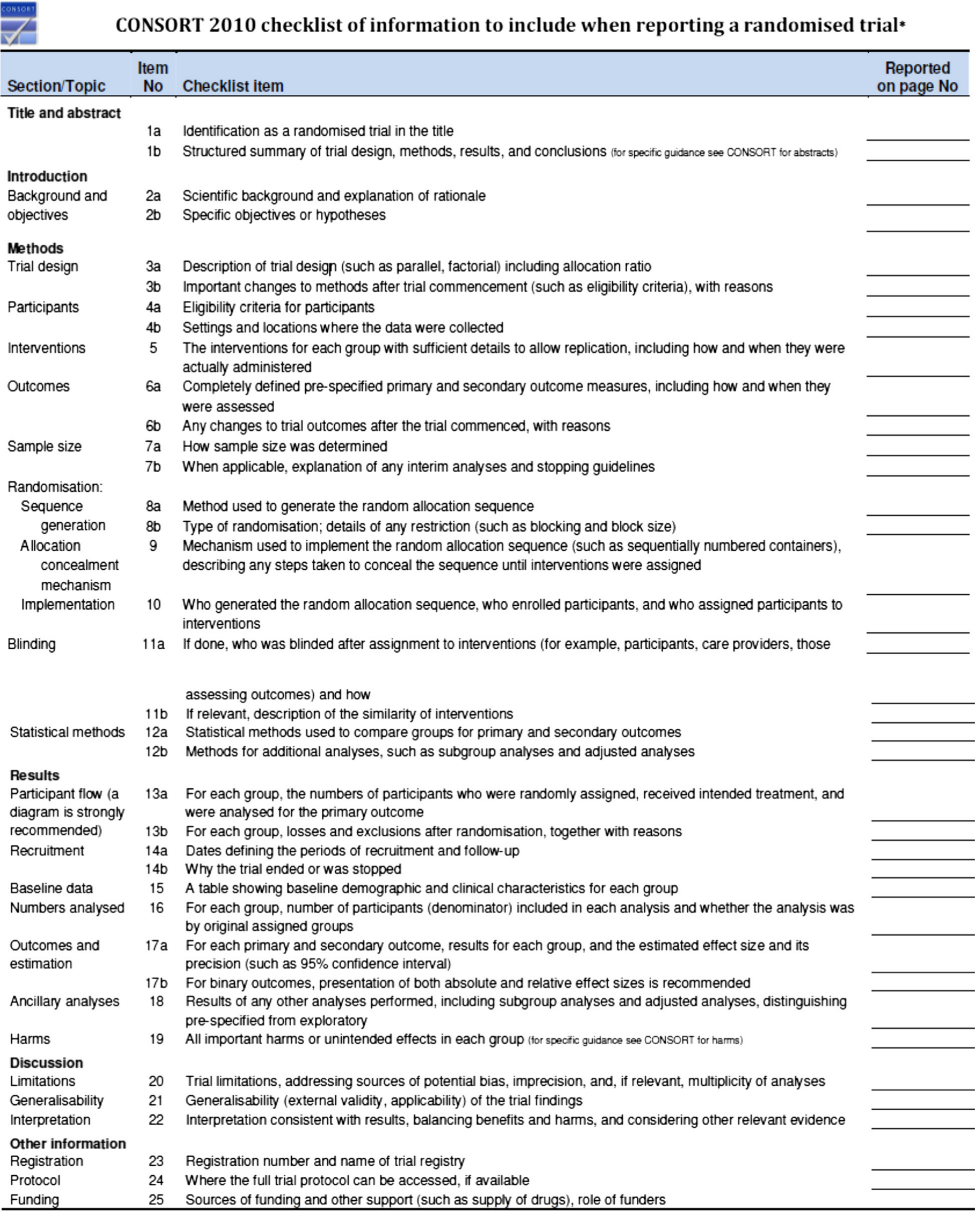
The CONSORT (CONsolidated Standards Of Reporting Trials) checklist, available at http://www.consort-statement.org/

Reporting guideline checklists follow the flow of the paper, presenting items in approximately the order encountered, from the Introduction through to Discussion (Fig. 2). Many, but not all journals require that authors provide a completed checklist for a submitted paper, indicating where each checklist item has been addressed. Some journals assess the checklist in the editorial office, others expect reviewers to. It is good practice for reviewers to examine the checklist, if provided, to ensure that all items have been adequately addressed. As well as being familiar with the checklist items, reviewers who are not familiar with the relevant reporting guideline may find it helpful to read the full explanation and elaboration paper that accompanies the reporting guideline to understand the rationale for each item and see examples of ‘good’ reporting.

Noting reporting guideline items that have not been reported, or not reported well, should be included in your review. You might detect a major flaw, such as inappropriate statistical analyses or something that has increased the study’s risk of bias but has not been appropriately explained or accounted for in the limitations and interpretations of the results. However, not adhering to a reporting item does not necessarily constitute a major flaw: missing information might simply reflect the author not reporting specific or enough information in the paper, something which might be easily resolved at the revision stage, and so might be more appropriate to classify as a minor flaw.

The journal that you are reviewing for might also have specific requirements. For example, the *BMJ* now requires a statement about patient involvement in setting the research question, and the design, implementation, and dissemination of the research [11]. These additional requirements will typically be communicated to you when the paper is sent to you for review.

### Reporting guidelines for RCTs

The CONsolidated Standards of Reporting Trials (CONSORT) Statement has been available for many years now (Checklist in Fig. 1). Over the years, a number of CONSORT extension statements have been developed [12]. Whether to use the main CONSORT statement or an extension depends on the RCT that you are reviewing. Some extensions are specific to the design of the RCT – for example, there is an extension for cluster RCTs [13] and one for pragmatic RCTs [14]. Others are specific to the type of data that were collected, such as the extension for reporting harms outcomes [15]. Other extensions are generic and are intended to be used with most RCTs, regardless of the design or data – for example, the Template for Intervention Description and Replication (TIDieR) guide, is an extension to item 5 of CONSORT and provides guidance for how to describe the intervention that was evaluated [16]. Familiarizing yourself with the CONSORT statement and its various extensions so that you know which to use during peer review is a good idea.

## Final thoughts

### Dealing with your own biases, prejudices, and conflicts of interest

Sometimes you will be asked to comment on a paper that is antithetical to your point of view. It is important that your own position does not make you overly critical – and similarly, papers that exactly reinforce your approach should not receive a less critical review. For many journals, you will be asked to complete a declaration of Conflict of Interest (also called, perhaps more accurately, a ‘Competing Interests’) form. You can list your prejudices here among the usual questions about whether you have financial or non-financial interests that might be advantaged or disadvantaged by publication of the paper. This will enable the Editor to evaluate your comments appropriately.

### Be nice

While the rejection of a paper can be demoralizing, all authors experience it at some stage and constructive reviews improve papers. Because publication in quality journals can influence careers in a very competitive world, rejection can exact a heavy toll. Reviewers have a responsibility to deliver criticisms carefully and constructively. There is usually something good about every paper that you can find to comment on. Making some positive and constructive comments to go with the disagreeable ones about aspects of the paper that need to be improved can help to keep your review balanced. Of course, positive comments must not be overdone in poor quality papers – this can create problems for the Editors if the Authors subsequently appeal: “*How can you reject my paper when…*”, or even “*Why should I revise it? ……the reviewers thought it was fine!*”

### Confidentiality and fully disclosed comments

Different journals have widely different policies. The usual system allows your comments, which are written anonymously, to be read by the submitting authors (who may also be anonymous), with an additional place to provide confidential comments that the paper authors do not see. This can be used to advise the editors about something very sensitive – for example, that you suspect plagiarism or research misconduct, or that you have some personal thoughts that you need to keep private for some reason. Otherwise, anything you have to say should be said transparently to the Authors just as much as the Editors.

Many journals now make the identity of the reviewer as well as the authors open (and not just to each other; some journals also post reviewers’ comments online alongside the final published article). But in any case, always imagine that your identity is known to the authors, and afford respect and courtesies accordingly. In other words, never hide behind anonymity (if that is the journal’s protocol) to write rude or *ad hominem* comments.

### Don’t be late

Many authors wait anxiously for news of their paper from the journal. It seems to take forever, and the delay is often very costly for them – they cannot easily progress with a second paper, they need the publication for promotion or a grant application, and so on. The rate-limiting step can sometimes be finding appropriate people who agree to review the paper and then waiting for the reviewers’ reports to be submitted. If you commit to doing the review, make sure you include the ‘complete-by’ date in that commitment. Many journals have elaborate reminder systems. Additional delay is as discourteous as a rude comment.

### Before hitting submit

We all have more to do than time to do it, and it can sometimes be tempted to rush the completion of a refereeing task. Check it for typographical and grammatical errors (it is particularly galling for an author to be criticized for typos from a review peppered with them), as well as general sense.

## Conclusions

The role of a reviewer comes with responsibility. The contribution that peer review can make to ensuring that published research is valid and clearly reported is often influenced by the quality of the peer review itself. Performing peer review of papers is an important contribution that every researcher needs to make, and it can be rewarding for a variety of reasons – including that, by reviewing papers, you learn to identify errors which you may then be less likely to make yourself. Developing solid skills in peer reviewing research papers is as important a skill as being able to write one.

## Box 1 Examples of major flaws that must be addressed before publication is possible

**Major flaws - research papers in general**

- Required ethics approval, and participant consent, was not obtained. This may be considered a fatal flaw by most journals;
- Methods that are not described sufficiently well to enable replication of the study;
- *Post hoc* analyses – that is, analyses that are undertaken after the data are collected and examined, but were not planned beforehand (*a priori*). The danger of doing this is that interesting analyses might be presented, while uninteresting ones are not, thereby introducing a bias. If performed, *post hoc* analyses should be carefully labelled as such to avoid confusion, or, only be presented as hypothesis-generating, to be formally tested in a future study;
- Important and relevant research not cited (especially studies with results that contradict the study being reviewed);
- Study limitations not adequately acknowledged;
- Conclusions that do not match the results and are not supported by data.

**Major flaws - RCTs**

- The trial was not prospectively registered in an appropriate trial registry, prior to the recruitment of participants. This is fatal for many (but not all) journals [17], and one alternative (not as ideal as prospective registration, but better than non-publication) is to deposit results with an acceptable registry. Mandating the registration of trials is designed to reduce bias in the literature that occurs from the differential publishing of studies with positive (or ‘interesting’) results rather than negative ones, leaving the literature (as reflected in systematic reviews, for example) with a positive bias [18]. It also prevents bias from the *post hoc* analyses of subgroups discussed in Box 1.
